# Metabolomics As a Tool for the Characterization of Drug-Resistant Epilepsy

**DOI:** 10.3389/fneur.2017.00459

**Published:** 2017-09-04

**Authors:** Federica Murgia, Antonella Muroni, Monica Puligheddu, Lorenzo Polizzi, Luigi Barberini, Gianni Orofino, Paolo Solla, Simone Poddighe, Francesco Del Carratore, Julian L. Griffin, Luigi Atzori, Francesco Marrosu

**Affiliations:** ^1^Department of Biomedical Science, University of Cagliari, Cagliari, Italy; ^2^Azienda Ospedaliera Universitaria (A.O.U) of Cagliari, Cagliari, Italy; ^3^Department of Medical Sciences and Public Health, University of Cagliari, Cagliari, Italy; ^4^Unité de Chimie Environnementale et Interactions sur le Vivant, Université du Littoral Côte d’Opale, Dunkerque, France; ^5^Faculty of Life Sciences, Manchester Institute of Biotechnology, University of Manchester, Manchester, United Kingdom; ^6^Department of Biochemistry, University of Cambridge, Cambridge, United Kingdom

**Keywords:** metabolomics, epilepsy, drug-resistant epilepsy, biomarkers, ketone bodies

## Abstract

**Purpose:**

Drug resistance is a critical issue in the treatment of epilepsy, contributing to clinical emergencies and increasing both serious social and economic burdens on the health system. The wide variety of potential drug combinations followed by often failed consecutive attempts to match drugs to an individual patient may mean that this treatment stage may last for years with suboptimal benefit to the patient. Given these challenges, it is valuable to explore the availability of new methodologies able to shorten the period of determining a rationale pharmacologic treatment. Metabolomics could provide such a tool to investigate possible markers of drug resistance in subjects with epilepsy.

**Methods:**

Blood samples were collected from (1) controls (C) (*n* = 35), (2) patients with epilepsy “responder” (R) (*n* = 18), and (3) patients with epilepsy “non-responder” (NR) (*n* = 17) to the drug therapy. The samples were analyzed using nuclear magnetic resonance spectroscopy, followed by multivariate statistical analysis.

**Key findings:**

A different metabolic profile based on metabolomics analysis of the serum was observed between C and patients with epilepsy and also between R and NR patients. It was possible to identify the discriminant metabolites for the three classes under investigation. Serum from patients with epilepsy were characterized by increased levels of 3-OH-butyrate, 2-OH-valerate, 2-OH-butyrate, acetoacetate, acetone, acetate, choline, alanine, glutamate, scyllo-inositol (C < R < NR), and decreased concentration of glucose, lactate, and citrate compared to C (C > R > NR).

**Significance:**

In conclusion, metabolomics may represent an important tool for discovery of differences between subjects affected by epilepsy responding or resistant to therapies and for the study of its pathophysiology, optimizing the therapeutic resources and the quality of life of patients.

## Introduction

Drug resistance is a critical issue in patients suffering from epilepsy. Indeed, a sufficient control of seizures is only obtained in half the population of patients with epilepsy, while in the remaining half, a subset of about 30% are classified as resistant to antiepileptic drugs (AEDs) (1). Although still debated, the definition of “drug resistance in epilepsy” can be broadly stated as “…a partial or no response to drugs that determines the presence of disabling seizures, which lead the affected individual to a significant neuropsychiatric and social impairment, lowering the quality of life, causing increased morbidity and higher risk of sudden death” (2). Moreover, it is worth noting that such clinical failures in controlling seizures correspond to a severe economic impact given that 6,000,000 patients are estimated in Europe alone as having active epilepsy, with an annual cost of € 20 billion euro (3), plus the associated familiar and social excruciating burden. Perhaps, one of the less discussed problems is represented by the urgency of determining the correct combination of drugs to treat epilepsy or indeed whether there is a combination of drugs that do indeed work for a given patient. This problem is well illustrated by the fact that the pursuit of the most efficient AEDs can take years to even attempt the ideal combination (4). Moreover, since most of these patients can be eligible for non-pharmacological or resective treatment or vagal nerve stimulation (VNS^®^ Cyberonics USA), there is an urgent need to find biomarkers that timely gage an individual’s drug resistance. To investigate this issue, in the present study, we have applied a metabolomics approach to identify the metabolic profile of AEDs pharmacoresistance. Metabolomics is an effective postgenomics research tool that, through the metabolic study of biological fluids, has been applied to many disciplines including the study of human diseases, food control, and plant physiology (5–7). In addition, metabolomics has recently been used for studying several neurological diseases (8–10). The study of biofluids has been associated with the use of several analytical techniques of separation and detection, including gas chromatography or liquid chromatography coupled to mass spectrometry and nuclear magnetic resonance (NMR) spectroscopy (11–13). Depending on the instrument used to generate the metabolomics profile, a different panel of metabolites is obtained. In particular, NMR is characterized by lower sensitivity, but higher reproducibility and relatively easy quantification compared to MS analysis. Following this approach, the metabolic phenotype of an individual can be characterized according to how the individual’s metabolism is influenced by many disparate factors such as genes, environment, nutrition, microbiota, and drugs. The application of metabolomics is an important step toward the understanding of the role that metabolic components have in disease and has been considered a real “*quantum leap*” advance in diagnosis, in staging, and, eventually, in categorizing different clusters of the same disease (14). On the basis of the results obtained in the present investigation, we suggest that the metabolomics approach is a viable new tool for neuroscience and in this case could help the clinician in the diagnosis of pharmacoresistance in epilepsy.

## Materials and Methods

### Subjects Selection

Subjects affected by epilepsy, either pharmacologically controlled or pharmacoresistant, were enrolled from patients monitored in the Epilepsy Diagnostic and Treatment Centre of the University of Cagliari (Italy), along with matched healthy controls (Table 1). The study was approved by the ethical committee of the University Hospital of Cagliari, and written informed consent was obtained from each patient before inclusion. Subjects enrolled were not on ketogenic diet (KD).

**Table 1:** Summary of the patients enrolled in the study: healthy controls, responders (R), and non-responders (NR) under therapy with AEDs.

Classes	Age (mean ± SD)/range	Gender (F/M)	Age at onset	Seiz/Trim	AEDs	Type of Seiz Foc/Gen	MRI N/SWMG
Controls (*n* = 35)	44.68 (±16.33)^a^/22–76	24/11	–	–	–	–	–
R (*n* = 18)	47.5 (±16.86)^a^/27–80	12/6	15.9 ± 5.3	2 ± 106	Under therapy	12/6	13/5
NR (*n* = 17)	52.17 (±9.57)^a^/41–71	11/6	15.4 ± 5.8	30 ± 12	Under therapy	11/6	13/4

*AEDs, antiepileptic drugs; Seiz/Trim, seizure at trimester; type of seizure, focal or generalized; MRI, magnetic resonance imaging; N, normal; SWMG, Small White Matter Gliosis*.

*^a^NS (*p* > 0.05)*.

As shown in Table 2, 17 individuals affected by drug-resistant, complex focal or generalized epilepsy were enrolled in the study. Among them, 15 subjects received AEDs in various combinations, while 2 in addition to AEDs had been implanted with a VNS device. The group of responders (R) was represented by 18 subjects, comparable with the AEDs-resistant group for type of seizures and mean age (Table 2) as well as for similar AEDs. Patients on monotherapy were not included in the study to better match the two epileptic groups. In subjects affected by focal seizures, the most probable type of seizure was determined both on the basis of reports from the patients themselves or from family members who witnessed events, as well as by ictal and interictal digital video-EEG and by 24-h EEG (Holter-EEG) recordings. On the basis of these criteria, the group of AEDs-resistant subjects included 6 patients with generalized epilepsy and 11 affected by focal seizures, while among the AEDs-responder patients, 6 suffered from generalized and 12 from focal seizures. No pathological signals were observed by magnetic resonance imaging (MRI) analysis. Small white matter altered microvascular spots were considered by neuroradiologist as age compatible (Table 2). The selection criteria included relative stability of clinical features related to interictal EEG activity, while the AEDs range in the serum was monthly assessed in optimal dosage in responder (R) and non-responder (NR) patients. The subjects affected by generalized and focal epilepsy were considered eligible if they had been monitored for more than 4 years. Further selection criteria for inclusion in the study were as follows: (i) stable clinical symptomatology and EEG features from the last three months; (ii) normal neurological examination and psychiatric evaluations according to DSM-V; (iii) recent brain MRI negative for potentially epileptogenic alterations (stroke, tumors, MAV, infectious diseases); and (iv) AEDs treatment in both pharmacoresistant and R groups given at stable dose from at least 3 months (Table 2). All selected patients did not receive KD. Seizure frequency occurrence was determined by the number of seizures reported in a personal diary (either by patient and/or caregivers). These data were standardized by considering the number of seizures during the 3-month period before the study; this period was arbitrarily selected (15). The selected subjects exhibited their first seizure between 8 and 26 years of age, and the seizure frequency of the AEDs-resistant subjects ranged from 14 to 52 episodes in the trimester preceding the day of the metabolomics study, while the AEDs controlled group presented from 0 to a maximum of 4 seizures in the trimester preceding the study (Table 2).

**Table 2:** Summary of the epileptic patients enrolled in the study: responders (R) and non-responders (NR) under therapy with AEDs.

	Patients	Gender	Age	Age at onset	Seiz/trimester	AEDs	Type of seiz	MRI
Responder	1 R	F	52	12	4	CBZ + TOP	FOC	N
	2 R	M	27	18	2	LMT + PB	GEN	N
	3 R	F	57	16	0	FELB + CBZ	FOC	N
	4 R	M	74	9	3	CBZ + LEV	FOC	N
	5 R	F	27	19	1	LEV + PB	GEN	N
	6 R	M	45	15	0	LMT + TOP + CBZ	FOC	SWMG
	7 R	F	76	11	1	CBZ + LMT	FOC	SWMG
	8 R	F	44	8	3	LMT + LEV + PB	GEN	SWMG
	9 R	F	43	16	1	OXC + TOP	FOC	N
	10 R	M	40	19	4	LMT + TOP	FOC	N
	11 R	F	44	24	2	CBZ + LEV	FOC	N
	12 R	M	80	9	0	LMT + LEV + PB	GEN	N
	13 R	M	67	21	1	OXC + LEV + CBZ	FOC	SWMG
	14 R	F	41	26	0	FELB + CBZ	FOC	N
	15 R	F	41	15	4	LMT + CBZ	FOC	N
	16 R	F	27	21	1	LMT + TOP	GEN	N
	17 R	F	37	12	5	OXC + LEV	FOC	SWMG
	18 R	F	33	22	3	CBZ + LMT	GEN	N

Non-responder	1 NR	F	41	14	34	CBZ + LEV	FOC	N
	2 NR	F	54	17	40	LMT + TOP	FOC	SWMG
	3 NR	F	42	23	38	CBZ + TOP + VNS	FOC	1NR
	4 NR	F	55	8	22	PRI + LEV + VNS	GEN	N
	5 NR	M	44	12	30	CBZ + FELB	FOC	N
	6 NR	M	44	20	52	OXC + LEV	FOC	SWMG
	7 NR	F	63	14	18	PB + CBZ	GEN	N
	8 NR	F	70	16	28	CBZ + LEV	FOC	N
	9 NR	F	71	18	12	LMT + FELB	FOC	N
	10 NR	M	46	9	38	CBZ + TOP	FOC	SWMG
	11 NR	F	48	11	32	PB + LEV + LMT	FOC	N
	12 NR	M	58	26	14	OXC + LEV	GEN	N
	13 NR	F	60	11	51	LMT + LEV + TOP	FOC	SWMG
	14 NR	M	48	16	22	PB + TOP	GEN	N
	15 NR	M	41	25	18	FELB + CBZ	GEN	N
	16 NR	F	49	8	24	CBZ + LEV	FOC	N
	17 NR	F	53	13	36	LMT + TOP + CBZ	GEN	N

*AEDs, antiepileptic drugs; CBZ, carbamazepine; LEV, levetiracetam; LMT, lamotrigine; TOP, topiramate; VNS, vagal nerve stimulation; FELB, felbamate; PB, phenobarbital; OXC, oxcarbazepine; PRI, primidone; type of seizure, focal or generalized; MRI, magnetic resonance imaging; N, normal; SWMG, Small White Matter Gliosis*.

The controls were matched by age and gender and were healthy subjects without neurological symptoms and chronic diseases.

Blood samples of patients and controls were collected after overnight fasting to minimize the immediate effects of the food.

Patients receiving valproic acid and lacosamide were excluded because the NMR resonances of these compounds affect the recognition of certain metabolites in the NMR spectra of serum.

### Samples Preparation and Acquisition

The blood samples were centrifuged for 10 min at 1,700 *g*, afterward the serum was obtained, carefully collected into aliquots of 1 ml, and stored at −80°C until analysis. All procedures related to samples preparation and acquisition of the data were performed according to internal standard protocols, previously published (16). Serum samples were thawed and centrifuged at 2,500 *g* for 10 min at 4°C. An aliquot of 800 µl of serum was used, and a solution of chloroform/methanol 1:1 (2,400 µl) plus 350 µl of distilled water was added. Samples were vortexed for 1 min and centrifuged for 30 min at 1,700 *g* at RT. After the centrifugation, hydrophilic and hydrophobic phases were collected. The first was concentrated overnight using a speed vacuum instrument (Eppendorf concentrator plus, Eppendorf AG, Hamburg, Germany) and then resuspended in 630 µl of D_2_O and 70 µl trimethylsilylpropanoic acid (TSP) 5.07 mM (f.c. = 0.507 mM). TSP was added to provide an internal reference for the chemical shifts of the spectrum obtained with the NMR analysis. Six hundred and fifty microliters of the solution were transferred into 5 mm NMR tubes. NMR experiments were acquired with a Varian UNITY INOVA 500 spectrometer (Agilent technologies, Inc., Santa Clara, CA, USA) operating at 499 MHz equipped with a 5 mm triple resonance probe with z-axis pulsed field gradients and autosampler. One-dimensional ^1^H-NMR spectra were collected at 300 K with a NOESY pulse sequence to suppress the residual signal of water by using 0.100 ms of mixing time. Spectra were recorded with a spectral width of 6,000, 2 Hz, acquisition time of 1.5 s, relaxation delay of 2 ms, 90° pulse of 9.2 µs, and number of scan of 256. Each free induction decay was zero-filled to 64K points and multiplied by a 0.5-Hz exponential line-broadening function. Spectra were manually phased, baseline corrected, and chemical shifts referred to the internal standard TSP (at δ = 0.0 ppm) using MestReNova software (version 8.1, Mestrelab Research S.L. Spain).

### Statistical Analysis

All spectra from NMR analysis were processed as previously reported (16). Each spectrum was divided into consecutive “bins” 0.04 ppm wide. The spectral area under investigation was the region between 0.6 and 8.6 ppm. To remove variations in the presaturation of the residual water resonance and spectral regions of noise, the regions between 4.64 and 5.2 ppm and between 5.28 and 6.6 ppm were excluded. The integrated area within each bin was normalized to percent values to minimize the effects of the different concentrations of serum samples. No interference from the drug metabolites, apart from valproic acid and lacosamide (which were excluded from analysis), was observed.

The final data set consisted of a 159 by 70 matrix (variables-bin × samples-subjects) values. Multivariate statistical analysis was performed on the generated matrix using SIMCA-P + software (version 13.0, Umetrics, Umeå, Sweden). NMR’s variables were scaled using Pareto scaling. Initial data analyses were conducted using principal components analysis (PCA). Then partial least square discriminant analysis and orthogonal partial least square discriminant analysis (OPLS-DA) were applied. The quality of the model was assessed with two parameters: R^2^ and Q^2^ cumulative. R^2^ estimates goodness of fit and Q^2^ estimates goodness of prediction. Each mathematical model was validated by permutation test. This test was used to validate the model and to assess the degree of overfitting, by means of *-n* random permutations (*n* = 500). The resulting plot displays the correlation coefficient between the original y-variable and the permuted y-variable vs the cumulative R^2^ and Q^2^, and the regression line. The scores values from each OPLS-DA model were subjected to CV-ANOVA to test the significance of the model, and the validation was considered successful with *p* < 0.05. Through the analysis of the S-plot, visualizing both the covariance and the correlation structure between the X-variables and the predictive score, and through the analysis of the set of VIPs (important variables on the projection), it was possible to identify the variables characterizing each class. These most significantly variables were quantified using Chenomx NMR Suite 7.1 and literature (17). A representative NMR spectrum showing the discriminate attribution is represented in Figure S1 in Supplementary Material. The concentrations were used to perform a non-parametric test, in particular, *U*-Mann–Whitney test, followed by a Holm–Bonferroni correction (GraphPad Prism software version 7.01, GraphPad Software, Inc., CA, USA).

## Results

**Figure 1:**
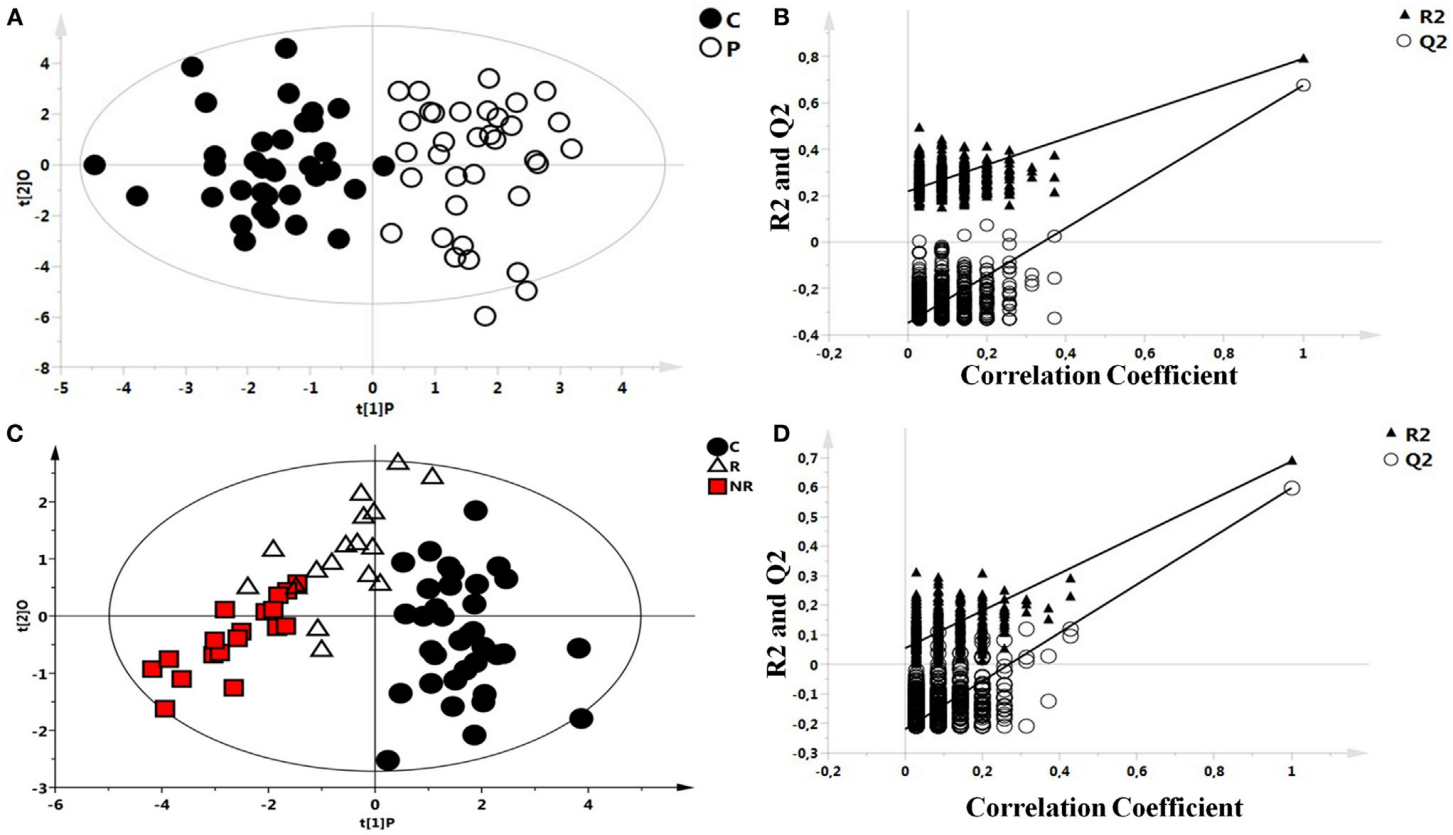
Scores plots obtained from nuclear magnetic resonance spectra of serum samples from controls and patients with epilepsy. **(A)** Scores plot from the multivariate orthogonal partial least square discriminant analysis model between controls (C): C (●) and patients with epilepsy: P (O): each point represents a single serum spectrum, with the position determined by the contribution of the 159 variables. **(B)** Validation of the corresponding model by permutation test (*n* = 500). **(C)** Scores plot from the multivariate orthogonal partial least square discriminant analysis of a three classes model: healthy subjects (●), responder (R) patients (Δ), and non-responder (NR) patients (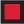). **(D)** Statistical validation of the corresponding model by permutation test.

As an initial step, the possible presence of outliers in our population was tested through the analysis of all the NMR spectra of blood serum by PCA. All samples passed this initial filtering step. Then, OPLS-DA was performed between the two groups and validated: controls and patients with epilepsy (Figures 1A,B). A significant different distribution of metabolites for the two groups was observed (*p* < 0.001). Model parameters for the explained variation (R^2^X and R^2^Y) and the predictive capability (Q^2^) were significantly high (R^2^X = 0.571; R^2^Y = 0.790; Q^2^ = 0.690), indicating robust classification of the two groups.

After observing a different metabolic profile between controls and patients with epilepsy, a possible difference between R and NR patients was investigated, too. OPLS-DA analysis of a three classes model, controls, R, and NR patients (Figure 1C), indicated a significant *p* value (*p* < 0.001) validated with the permutation test (Figure 1D).

Statistical parameters of this model were R^2^X = 0.664, R^2^Y = 0.615, and Q^2^ = 0.488. Validation of the model was performed by permutation test.

**Figure 2:**
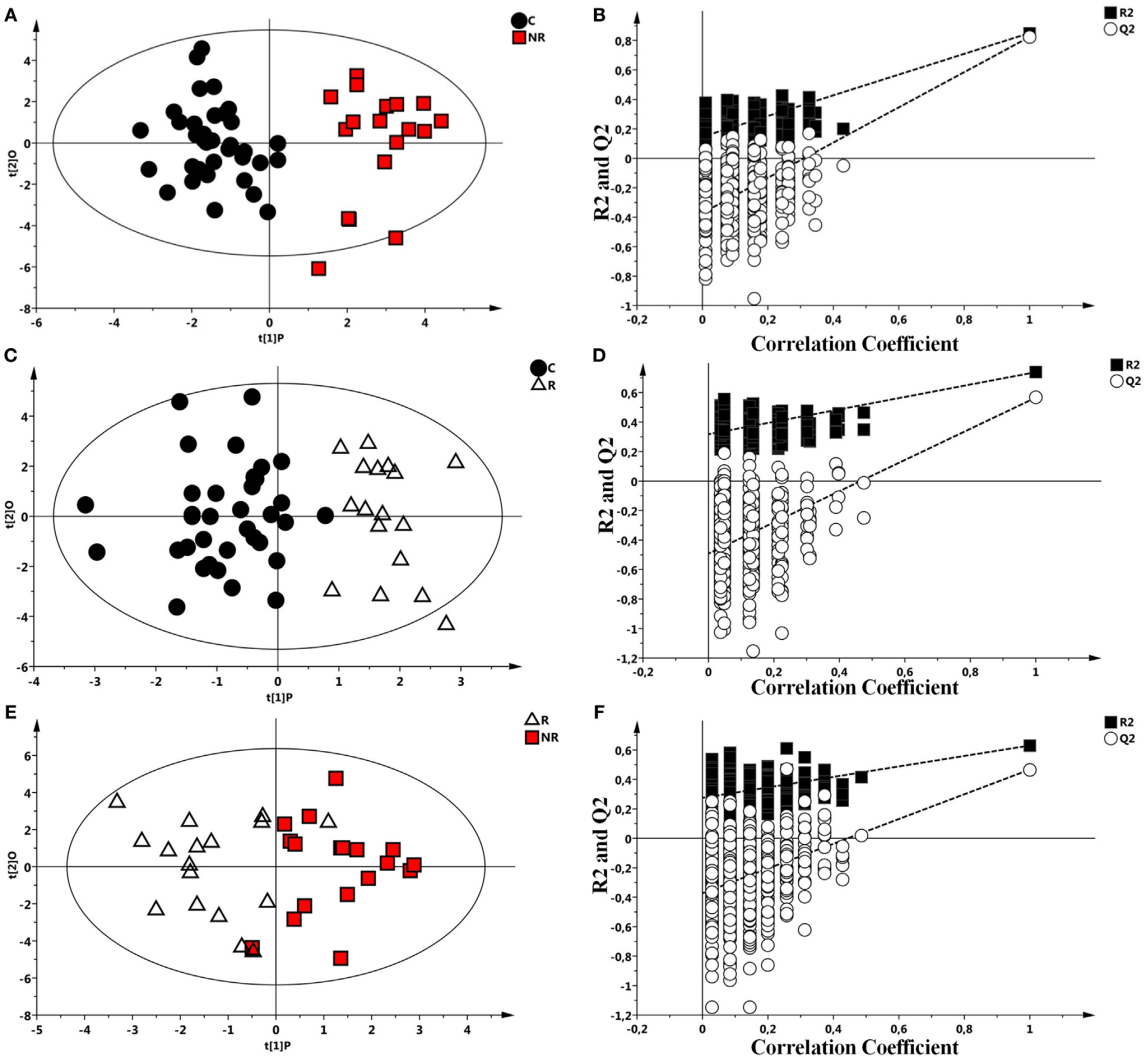
Scores plots obtained from nuclear magnetic resonance spectra of serum samples from controls and responder (R) and non-responder (NR) patients. **(A)** Scores plot from the multivariate orthogonal partial least square discriminant analysis (OPLS-DA) model between controls (●) and NR patients (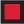). **(B)** Statistical validation of the corresponding model by permutation test (*n* = 500). **(C)** OPLS-DA between controls (●) and R patient (Δ) and **(D)** statistical validation of the corresponding model by permutation test (*n* = 500). **(E)** OPLS-DA model between R (Δ) and NR patients (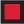). **(F)** Statistical validation of the corresponding model by permutation test (*n* = 500).

Each class was individually compared with the others by OPLS-DA and results validated by a permutation test (Figures 2A–F). All the analysis showed significant statistical values positive both in terms of variance of predictive capability and *p* value. Statistical parameters are reported in Table 3.

**Table 3:** Summary of the statistical parameters of the models C vs non-responder (NR), C vs responder (R), and R vs NR.

	Orthogonal partial least square discriminant analysis models					Permutation^d^	
Groups	Components^a^	R^2^Xcum^b^	R^2^Ycum^b^	Q^2^cum^c^	*p*	R^2^ intercept	Q^2^ intercept
Controls vs NR	1P + 1O	0.518	0.850	0.824	<0.001	0.140	−0.360
Controls vs R	1P + 1O	0.545	0.739	0.566	<0.001	0.316	−0.490
Responders vs NR	1P + 1O	0.508	0.631	0.467	<0.001	0.278	−0.395

*^a^The number of predictive and orthogonal components used to create the statistical models*.

*^b^R^2^X and R^2^Y indicated the cumulative explained fraction of the variation of the X block and Y block for the extracted components*.

*^c^Q^2^cum values indicated cumulative predicted fraction of the variation of the Y block for the extracted components*.

*^d^R^2^ and Q^2^ intercept values are indicative of the validity of the model. The permutation test was evaluated on the corresponding partial least square discriminant analysis model*.

For each OPLS-DA model, it was possible to identify the discriminant metabolites for the three classes under investigation. The metabolites corresponding to the discriminants variables were identified and quantified by using Chenomx software. Metabolic discriminants were similar among the different groups. Serum from epileptics patients were characterized by increased levels of 3-OH-butyrate, 2-OH-valerate, 2-OH-butyrate, acetoacetate, acetone, acetate, choline, alanine, glutamate, and scyllo-inositol (C < R < NR) and decreased concentration of glucose, lactate, and citrate compared to controls (C > R > NR) as showed in Table S1 in Supplementary Material.

The matrix containing the concentrations of discriminant metabolites for each patient belonging to the three groups was analyzed to carry out an analysis of the variance with multivariate and univariate tests. The multivariate analysis showed that the discriminating metabolites of each mathematical models previously analyzed were similar. Moreover, this analysis showed differences in terms of concentration depending on the classes, suggesting a possible role of the therapeutic effect in R patients. 2-OH-butyrate and 2-OH-valerate were not quantified because signals overlapped in the same spectral region (triplet at 0.92 ppm). Univariate analysis using metabolite concentrations as variable was conducted on all groups. *U*-Mann–Whitney test was used to explore and compare the mean differences between the groups (Table 4), and then Holm–Bonferroni correction was applied. The results are showed in Table 4.

**Table 4:** Metabolites significant altered among the classes control (C), responder (R), and non-responder (NR).

Metabolites	C mean (mM) ± SD	R mean (mM) ± SD	NR mean (mM) ± SD	*p* value [Mann–Whitney test (MW) test]: C vs R	*p* value [Holm–Bonferroni correction (HBonf.C)]: C vs R	*p* value (MW test): C vs NR	*p* value (HBonf.C): C vs NR	*p* value (MW test): R vs NR	*p* value (HBonf.C): R vs NR
3-OH-butyrate	0.10 ± 0.07	0.12 ± 0.06	0.14 ± 0.1	ns	ns	0.002	0.01	ns	Ns
Acetate	0.061 ± 0.01	0.09 ± 0.02	0.1 ± 0.02	<0.0001	0.001	0.002	0.01	ns	ns
Acetoacetate	0.01 ± 0.01	0.02 ± 0.01	0.03 ± 0.01	0.005	0.025	<0.0001	0.001	0.01	ns
Acetone	0.0008 ± 0.001	0.008 ± 0.008	0.02 ± 0.01	<0.0001	0.001	<0.0001	0.001	ns	ns
Citrate	0.12 ± 0.04	0.10 ± 0.04	0.09 ± 0.02	ns	ns	0.009	0.04	ns	ns
Glucose	2.02 ± 0.05	1.81 ± 0.03	1.74 ± 0.03	ns	ns	0.01	0.04	ns	ns
Lactate	1.88 ± 0.04	1.72 ± 0.05	1.16 ± 0.05	ns	ns	<0.001	0.001	0.001	0.007
Scyllo-inositol	0.16 ± 0.01	0.4 ± 0.5	0.24 ± 0.2	0.04	ns	ns	ns	ns	ns

*Summary of the univariate statistical analysis. U-MW was performed, and all the results underwent the HBonf.C*.

A different metabolic profile was identified for the three different groups; in particular the group of the patients with epilepsy was characterized by an increase of acetate, acetoacetate, acetone, and scyllo-inositol (R in particular) with respect to the control group, while it showed a decrease of lactate, glucose, and citrate.

## Discussion

The present study in patients with drug-resistant epilepsy shows that the concentrations of glucose, citrate, and lactate are decreased and ketone bodies (3-OH-butyrate, acetate, acetoacetate, and acetone) increased compared to R patients with epilepsy and controls. At first glance, our result appears in line with the observations that, in cases of refractory epilepsy, the energetic failure is reflected by uncompensated brain glucose levels (18). On the other hand, the increased concentrations of KBs in NR patients seem unexpected and at variance with the traditional studies that demonstrated the efficacy of the KD in controlling pharmacoresistant seizures (19). However, the key role of the KD, which represents the largest source of KBs, in the treatment of severe forms of infantile epilepsy (20) as well as in adult seizures (21, 22) is still challenging (23–25). According to our data, subjects affected by frequent and intractable seizures show an increase of KBs compared to patients pharmacologically controlled, despite being treated with similar dose of AEDs (Table 2). The reduction of citrate, glucose, and lactate concentrations in NR patients compared with R and C patients suggest a switch from glucose metabolism, the suitable energetic substrate of the brain, to ketogenic metabolites. Indeed, previous studies reported similar variations including a reduction in ^13^C-labeled citrate in an experimental seizure model in the hippocampus (26, 27). It is also worth noting that in these studies the concentrations of citrate and glucose showed a parallel decrease similar to the lactate profile. These observations suggest that in conditions of frequent/uncontrolled convulsions the biochemical response of the cellular machinery might be forced toward alternative energy resources such as those derived from KBs utilization. The antiseizure effects induced by KD suggest several mechanisms encompassing bioenergetics and mitochondrial changes (28), activity of cellular oxidation *via* poly-unsaturated fatty acids (29), and regulation of neuroprotective factors (30–32). Moreover, as glutamate and γ-aminobutyric acid (GABA), respectively, are the major excitatory and inhibitory neurotransmitters in the brain several studies focused their main issues on how KBs affect levels of these neurotransmitters and their receptor activity (33). Following magnetic resonance spectroscopy investigations, it has been demonstrated that increased levels of GABA neurotransmission are detected in cerebrospinal fluid of patients with KD (34, 35) underlining a possible action of ketone bodies upon the modulation of GABA-A receptors by increasing the synaptic inhibition (35, 36). The increase of synaptic inhibition might not be *per se* sufficient in contrasting AEDs-resistant epileptogenesis since the brain networks under sustained seizure activity show a poor affinity of GABA receptors (37, 38). Moreover, several alternative mechanisms for the KD effects have been proposed recently. The antiepileptic action of medium-chain triglyceride, a good source of KBs, has been widely documented (39, 40). Recently, Chang et al. demonstrate that medium-chain triglyceride, rich in decanoic acid, provides an antiseizure effect acting through direct AMPA receptor inhibition (41).

Finally, the high production of KBs in patients with frequent seizure could be important to balance the increase of glutamate through the modification of the behavior of vesicular glutamate transporters (42). Together the effects of KBs emerge as a complex pleiotropic mechanism, which contains an apparent paradox: while the KBs are the most relevant part of a KD; nonetheless, their antiepileptic effects still needs to be proved (31). It seems possible to suggest that the increase of KBs in patients with epilepsy can be interpreted as an attempt to activate spontaneous biochemical processes aimed to optimize ultimate energetic resources.

The results of the present study represent an interesting finding that can help the clinician in evaluating subjects affected by severe epilepsy as potential pharmacoresistant patients, thus prompting new investigations addressed to find alternative therapeutic solutions. However, several lines of criticism emerge in commenting the present data. Among them (i) we cannot rule out that the population of R and NR might show a metabolomic pattern related to the level of seizure control and drug sensitivity; (ii) despite supporting data also from the literature, we cannot point at the metabolomic profile as the sole predictor mechanism of anticonvulsant effects, given the manifold players of epileptogenesis and pharmacoresistance (43, 44); (iii) there are no sufficient data that a possible seizure control might reverberate in a given metabolomic pattern; (iv) the metabolomic profile described in the present study has been derived from a single sample of each individual, while possible variations (e.g., related with seizure frequency or time of seizure) can be better assessed by multiple samples.

In conclusion, the results suggest that metabolomic can help to better understand some aspects of pharmacoresistant epilepsy and contribute to target some peculiar biochemical mechanisms involved in the epileptogenesis. A larger cohort of selected patients affected by drug-resistant epilepsy and a multicentric carefully planned study are needed to validate the present findings.

## Ethics Statement

This study was carried out in accordance with the recommendations of Ethical Committee of the University Hospital of Cagliari with written informed consent from all subjects. All subjects gave written informed consent in accordance with the Declaration of Helsinki. The protocol was approved by the Ethical Committee of the University Hospital of Cagliari.

## Author Contributions

LA and FM: equally contributing authors. LA, JLG and FM: conceived the study, directed the project and designed the experiments, and contributed to the final version. FM and SP: performed experiments and data analysis, performed metabolomics experiments and data analysis, wrote the first draft of the manuscript, critically reviewed the data and the manuscript, and read and approved the final version of the manuscript. AM, MP, LO, GO, and PS: obtained the samples and clinical details and performed data analysis. LB and FC: performed metabolomics experiments and data analysis. All authors critically reviewed the data and the manuscript. All authors read and approved the final version of the manuscript.

## Conflict of Interest Statement

The authors declare that the research was conducted in the absence of any commercial or financial relationships that could be construed as a potential conflict of interest.

## Supplementary Material

Figure S1 Assignments in an nuclear magnetic resonance spectrum of the discriminant metabolites resulting from the groups [control (C), responder (R), and non-responder (NR)] comparison. (1) 3-OH-butyrate; (2) lactate; (3) alanine; (4) acetate; (5) acetone; (6) acetoacetate;(7) glutamate; (8) citrate; (9) choline; (10) glucose; (11) scyllo-Inositol.
