# Supplementary figures and images for: Metabolomics As a Tool for the Characterization of Drug-Resistant Epilepsy
Source: Front Neurol. 2017 Sep 4;8:459. doi: 10.3389/fneur.2017.00459 (PMC5591409; doi:10.3389/fneur.2017.00459)

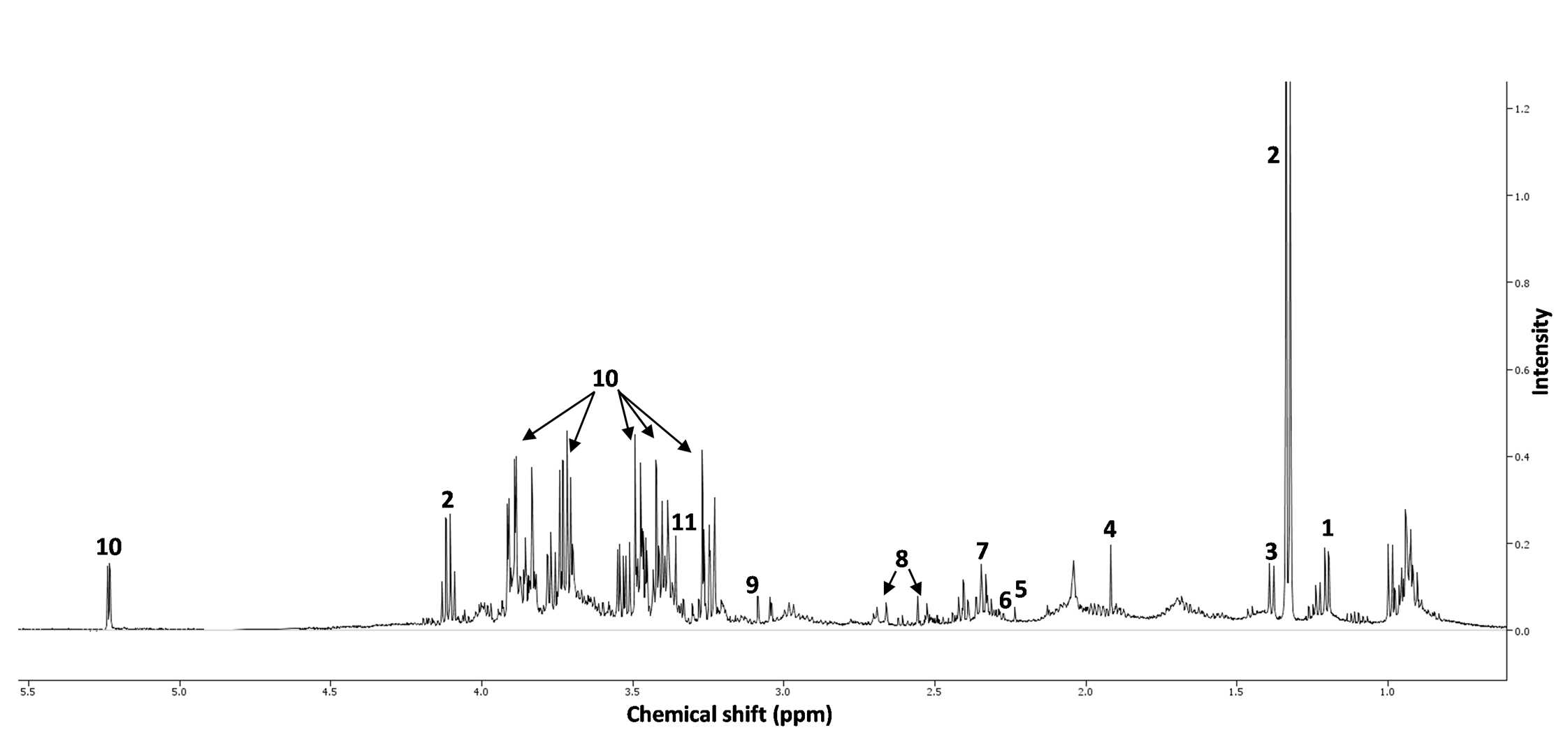

Supplement: Figure S1 — Assignments in an nuclear magnetic resonance spectrum of the discriminant metabolites resulting from the groups [control (C), responder (R), and non-responder (NR)] comparison. (1) 3-OH-butyrate; (2) lactate; (3) alanine; (4) acetate; (5) acetone; (6) acetoacetate;(7) glutamate; (8) citrate; (9) choline; (10) glucose; (11) scyllo-Inositol. [file image_1.tif]
