# Supplementary material for: Metabolomics As a Tool for the Characterization of Drug-Resistant Epilepsy
Source: Front Neurol. 2017 Sep 4;8:459. doi: 10.3389/fneur.2017.00459 (PMC5591409; doi:10.3389/fneur.2017.00459)
Supplement: Supplementary file 2 [file table_1.pdf]

Table 1S. Comparative table of the discriminant metabolites resulting from the analysis of the S-plot and VIP list of the multivariate models (C *vs* R, C *vs* NR and R *vs* NR).Amount of + indicates the class with higher or lower concentration of the different metabolites.

**Metabolites**	**C**	**R**	**NR**
**Citrate**	+++	++	+
**Lactate**	+++	++	+
**Glucose**	+++	++	+
**Glutamate**	++	+	++
**Scyllo-Inositol**	+	+++	+
**2-OH-Butyr/Valerate**	+	++	+++
**3-OH-Butyrate**	+	++	+++
**Acetoacetate**	+	++	+++
**Acetate**	+	++	+++
**Acetone**	+	++	+++
**Alanine**	+	+	++
**Choline**	+	++	++
